# High-Quality Fiber Bragg Gratings Inscribed by Femtosecond Laser Point-by-Point Technology

**DOI:** 10.3390/mi13111808

**Published:** 2022-10-23

**Authors:** Runxiao Chen, Jun He, Xizhen Xu, Jiafeng Wu, Ying Wang, Yiping Wang

**Affiliations:** 1 Key Laboratory of Optoelectronic Devices and Systems of Ministry of Education/Guangdong Province, College of Physics and Optoelectronic Engineering, Shenzhen University, Shenzhen 518060, China; 2 Shenzhen Key Laboratory of Photonic Devices and Sensing Systems for Internet of Things, Guangdong and Hong Kong Joint Research Centre for Optical Fibre Sensors, Shenzhen University, Shenzhen 518060, China

**Keywords:** Fiber Bragg gratings, femtosecond laser micromachining, point-by-point technology

## Abstract

We experimentally studied the inscription of fiber Bragg gratings by using femtosecond (*fs*) laser point-by-point (PbP) technology. The effects of the focusing geometry, grating order, laser energy and grating length on the spectral characteristics of the PbP FBG were investigated. After optimizing these parameters, a high-quality first-order PbP FBG with a reflectivity > 99.9% (i.e., Bragg resonance attenuation of 37.7 dB) and insertion loss (IL) of 0.03 dB was successfully created. Moreover, taking advantage of the excellent flexibility of the *fs* laser PbP technology, high-quality FBGs with various Bragg wavelengths ranging from 856 to 1902.6 nm were inscribed. Furthermore, wavelength-division-multiplexed (WDM) FBG arrays consisting of 10 FBGs were rapidly constructed. Additionally, a Fabry-Perot cavity was realized by using two high-quality FBGs, and its birefringence could be reduced from 3.04 × 10^−5^ to 1.77 × 10^−6^ by using a slit beam shaping-assisted femtosecond laser PbP technology. Therefore, such high-quality FBGs are promising to improve the performance of optical fiber sensors, lasers and communication devices.

## 1. Introduction

Fiber Bragg gratings (FBGs) have become an indispensable component element in modern optics and information technology since the first FBG proposed by Hill et al. in 1978 [1]. FBGs have played an important role in filters, wavelength-division-multiplexing (WDM), sensing, and laser cavities [2,3,4,5,6]. In particular, high-quality FBGs, characterized by high reflectivity, low insertion loss (IL) and low birefringence, have attracted great interest in recent years. Numerous research efforts have been dedicated to improving the performance of FBGs.

To date, three typical approaches are used to inscribe FBGs: the phase-mask technologies, laser Talbot interferometer methods, and femtosecond (*fs*) laser direct writing technologies. UV laser-based phase-mask technology is the most conventional method for the fabrication of FBGs, which can offer FBGs with high reflectivity and negligible loss [7]. Nevertheless, such a method requires additional hydrogen loading to enhance the photosensitivity of the fiber. In 2003, an *fs* laser phase-mask technology was proposed by Mihailov et al. [8]. The FBGs could be directly inscribed in non-photosensitive fibers in this method, presenting similar spectral characteristics with the UV laser-based phase-mask technology, as well as an enhanced temperature-resistant performance of 1000 °C [9]. The Bragg wavelength can be tuned by means of the application of strain to the optical fiber during inscription; however, the range is still limited. This phase-mask technology exhibits the inflexibility of wavelength-tunability and complex gratings design. Additionally, it is also complex and costly to inscribe FBG wavelength-division-multiplexed arrays, since it requires several phase masks with various periods. Alternatively, *fs* laser Talbot interferometer methods exhibit good wavelength flexibility for FBG inscription [10]. The period of the space interference pattern can be tuned by changing the laser incident angle (i.e., rotating the rotator mirrors), and thus producing FBGs with various grating periods. However, due to the poor temporal and spatial coherence properties of the *fs* laser, complicated adjustment of the space optical path is required, which shows low efficiency in FBG inscription.

The *fs* laser direct writing technologies have received much attention in recent years, since these technologies do not require a phase mask, and the grating period, shape and position of refractive index modulation (RIM) can be flexibly controlled [11]. Thus, FBGs with various Bragg wavelengths and complex spectra can be achieved [12]. The line-by-line (LbL) technology was first reported by Zhou et al. [13], in which line-shape RIMs were induced in the fiber core, leading to a 4.4 mm-long FBG with Bragg resonance attenuation of 17 dB and IL of 0.5 dB. Such an FBG had a low ratio of the coupling strength coefficient to the scattering loss coefficient *κ/α* = 46 (*κ* is the coupling strength coefficient and *α* is the scattering loss coefficient). To increase the κ/α, Williams et al. proposed a continuous core-scanning technique [14], achieving a first-order FBG with a Bragg resonance attenuation of 49 dB and IL of 0.1 dB, corresponding to a much higher *κ/α* > 780. Nevertheless, both the LbL and continuous core-scanning technologies are time-consuming in grating inscription. As the most common direct writing technology, point-by-point (PbP) technology was first reported by Marshall et al. [15]. This technology has the same flexibility as the other two technologies but a much higher efficiency in grating inscription. However, FBGs inscribed by PbP technology typically show a low *κ/α* (i.e., <100) and high birefringence due to the highly localized and elliptical RIMs in the fiber core [16,17]. Recently, *fs* laser beam shaping by a cylindrical lens or a slit was demonstrated to effectively increase the *κ/α* of point-by-point FBGs (PbP FBGs) [18,19]. We have employed the slit beam shaping method for creating high-quality FBGs with a *κ/α* of 172.46 [20]. Additionally, the intrinsic birefringence of the PbP FBGs could be effectively reduced by modulating the ellipticity of cross-sectional patterns of RIMs using a slit.

In this article, we further developed the *fs* laser PbP technology to inscribe high-quality FBGs. The effects of focusing geometry, grating order, laser energy and grating length on the spectral response were investigated. After optimizing these parameters, a high-quality first-order PbP FBG with a Bragg resonance attenuation of 37.7 dB and IL of 0.03 dB was successfully created. Additionally, high-quality FBGs with various Bragg wavelengths and WDM FBG arrays were flexibly created by the *fs* laser PbP technology. Moreover, the birefringence of the FBG Fabry-Perot (FBG-FP) cavity was investigated, and a slit beam shaping-assisted *fs* laser PbP technology was used, achieving an FBG-FP cavity with a low birefringence of 1.77 × 10^−6^. The inscribed high-quality FBGs are promising candidates for applications in optical fiber communications, sensing and lasers.

## 2. FBG Fabrication and Working Principle

**Figure 1:**
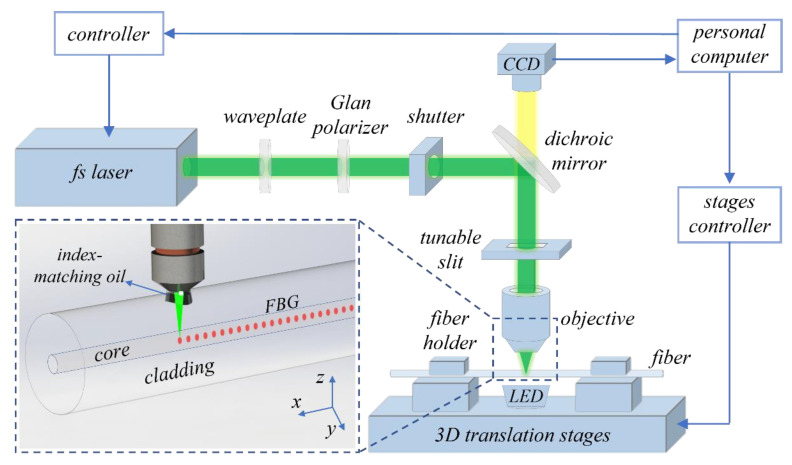
Experimental setup for inscribing FBG using *fs* laser PbP technology.

The experimental layout of the femtosecond laser micromachining setup for fabrication of the high-quality FBGs is shown in Figure 1. A frequency-doubled femtosecond laser amplifier (Light Conversion, Pharos PH1-10, Vilnius, Lithuania) with a central wavelength of 513 nm, a pulse duration of 290 *fs*, and a repetition of 200 kHz is used as the laser source. A waveplate and Glan polarizer are used to adjust the pulse energy and polarization of the laser beam. In particular, a tunable slit is inserted before the objective, which can modify the laser beam to the objective by changing the width of the slit. The laser beam is tightly focused in the core of a coating-stripped single mode fiber (SMF, Corning SMF-28, New York, NY, USA) by a high numerical aperture (NA) oil-immersion microscope objective. Index-matching oil is applied between the objective and the interface of the fiber for the purpose of reducing the aberration at the focal region. The fiber held on two clamps is mounted on a computer-controlled three-dimensional air bearing translation stage (X: ABL10100-LN; Y: ABL10100-LN; Z: ANT130V 5-CN1-PL2, Aerotech, Pittsburgh, KS, USA), ensuring the accurate translation of the fiber during the fabrication process. The microscopic image of the fiber is monitored in real time by a high-resolution CCD. During the FBG inscription process, the shutter is opened and the fiber was translated along the fiber axis (i.e., the x-axis in Figure 1), and thusly the focused laser pulses induce periodical refractive index modulation in the fiber core, thereby creating an FBG. The Bragg wavelength of the inscribed FBG is expressed as:

*mλ_B_* = *2 n_eff_Λ* (1)

where *λ_B_* is the Bragg wavelength, *n_eff_* is the effective refractive index of the fiber core, *m* is the order of the grating, and Λ is the grating period, which is determined by the velocity of the fiber translation stage *v* and the pulse repetition frequency of the *fs* laser *f*. By adjusting *v* or *f*, the FBG period can be flexibly changed through *Λ = v/f*. Note that the repetition translation *f* is set as 1 kHz by a frequency divider. Thus, it only takes several seconds to inscribe an FBG. The transmission and reflection spectra are recorded via a broadband light source (YSL, SC series, Wuhan, China), an optical spectrum analyzer (OSA, Yokogawa AQ6370C, Tokyo, Japan) with a resolution of 20 pm. For a uniform FBG, the reflectivity *R* can be expressed as:

*R* = *tanh*^2^(*κL*) (2)

where *κ* is the grating coupling strength coefficient, which is determined by the refractive index modulation (Δ*n*), mode overlap factor *η*, and Bragg wavelength *λ_B_*:

*κ* = *π*Δ*nη/λ_B_* (3)

The coupling strength coefficient *κ* and insertion loss (IL) of an PbP FBG are largely dependent on the size, shape and morphology of the refractive index modulations (RIMs) in the fiber core. The Δ*n* is the amplitude (or AC component) of the RIM, which could be controlled by changing the pulse energy and ratio of the modification to space in a grating pitch (i.e., duty cycle). Thus, to achieve a high-quality PbP FBG, one could tailor the focusing geometry, grating order, pulse energy and grating length. As for the birefringence of the PbP FBGs, it is highly dependent on the size, ellipticity and position of the RIMs in the fiber core [21], which could be modulated by spatial beam shaping of the *fs* laser beam.

## 3. Experimental Results

### 3.1. Effect of Objective NA on RIM and Spectral Response

The spectral quality of the FBG by *fs* laser point-by-point technology depends largely on the focusing conditions. In particular, the NA of the objective plays an important role in the fabrication of FBGs. If an *fs* laser beam with a central wavelength of *λ* is focused by a certain NA microscope objective into a fiber, the focused beam parameters can be expressed as:

*w*_0_ = *λ/π*NA, *z*_0_ = *nλ/π*NA^2^ (4)

where *w*_0_ and *z*_0_ are the beam waist and Rayleigh length of the focused beam, respectively, *n* is the effective refractive index of the fiber cladding. Note that both *w_0_* and *z_0_* are increasing as the NA of the objective decreases. That means the size of the RIM induced by *fs* laser pulse will change with objectives of various NA.

**Figure 2:**
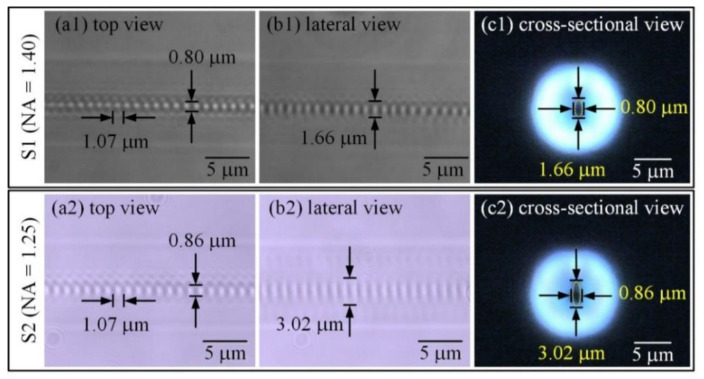
Grating morphologies inscribed with objective NA of 1.40 and 1.25, respectively. (**a**) Top-view, (**b**) side-view and (**c**) cross-sectional-view microscope images of FBGs inscribed with objective NA of 1.40 and 1.25.

Initially, we experimentally studied the effect of objective NA on the spectral response and RIM morphologies of the PbP FBGs. Herein, two 4 mm-long gratings were inscribed in two sections of an SMF by using two oil-immersion objectives with NAs of 1.40 and 1.25, respectively. The translation speed was set as 1.07 mm/sec and thus two FBGs with the same period of 1.070 μm were obtained. The on-target pulse energies for fabricating the gratings were 70 and 55 nJ, respectively. Note that it only takes ~3.7 s for the inscription of each FBG. To obtain the FBG cross-sectional morphologies, we firstly transversally cleaved the FBG at the grating region by a fiber cleaver (Sumitomo, FC-6S, Osaka, Japan). Then, a microscope (Leica, DM2700MH, Wetzlar, Germany) with an oil-immersion objective (Leica, 100×, numerical aperture: 1.32) was employed to observe the cross-sectional images. As a result, the cross-sectional images of the fiber core could be visualized and captured. Figure 2(a1,c1) and Figure 2(a2,c2) show the top-view, side-view and cross-sectional-view of the microscope images of the two FBGs inscribed by using objectives with 1.40 and 1.25 NA, respectively. We can observe that the RIM morphologies focused by 1.40 and 1.25 NA are elliptical micro-voids, which are induced by the nonlinear absorption and avalanche ionization of the tightly focused *fs* laser pulses in the fiber core [22]. In addition, the width of the RIM morphologies for 1.40- and 1.25-NA focusing are 0.80 and 0.86 μm, and the sizes along the beam propagation direction are 1.66 and 3.02 μm, respectively. Note that the RIM induced by 1.25-NA focusing was significantly larger than 1.40-NA focusing, in accordance with the theory of Equation (1). The mode overlap factor *η* is increased with the larger RIM, thus improving the coupling strength coefficient *κ* of grating.

**Figure 3:**
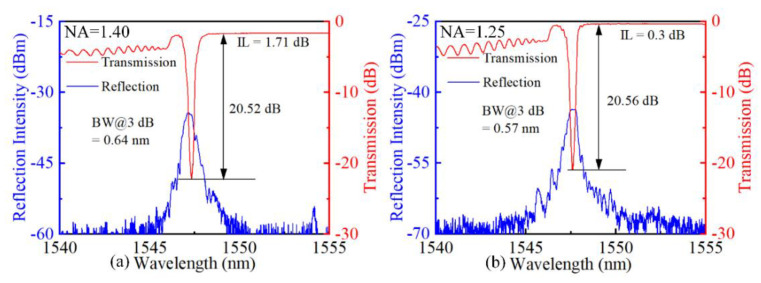
Transmission and reflection spectra inscribed with objective NA of (**a**) 1.40 and (**b**) 1.25.

Figure 3a,b shows the spectral responses of the inscribed FBGs. According to the results, the attenuations of Bragg resonance of the two FBGs are almost the same (i.e., 20.52 and 20.56 dB), the 3 dB bandwidths [i.e., full-width-at-half-maximum (FWHM)] are 0.64 and 0.57 nm, and the ILs are 1.7 and 0.3 dB, respectively. Notably, the FBG inscribed using a 1.25 NA objective shows a significantly lower insertion loss. Compared with the relatively large RIM induced by *fs* laser with a 1.25-NA focusing, the *fs* laser focused by the 1.40-NA objective led to a more localized RIM. Thus, for the fabrication of two FBGs with the same grating strength, a higher pulse energy was required to increase the RIM area while using the 1.40-NA objective. Then, an enhanced scattering loss was induced by the higher *fs* laser pulse energy focused by the higher NA objective [23]. As a result, the inscribed FBG focusing with a 1.25-NA oil-immersion objective exhibited a lower IL and a lager RIM, which is more suitable for fabricating high-quality FBGs. Therefore, the following investigations in the inscription of PbP FBGs are based on the objective with 1.25 NA.

### 3.2. Effect of Grating Order on Spectral Response

**Figure 4:**
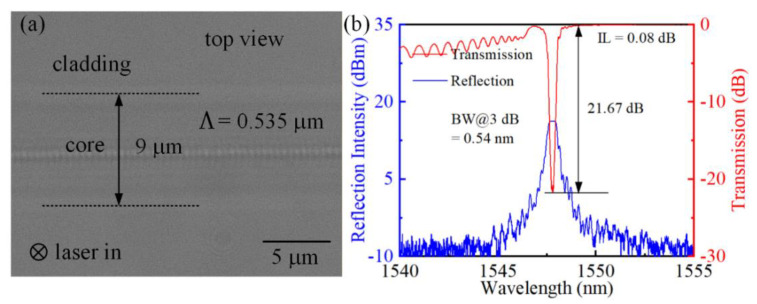
(**a**) Top-view morphology of the inscribed first-order FBG and (**b**) corresponding transmission and reflection spectra.

Next, we investigated the grating order on the FBG spectral response, since the grating order has a significant impact on the RIM Δ*n*. To investigate the effect of grating order, a 4 mm-long grating was inscribed using a translation speed of 0.535 mm/s. Notably, an optimized pulse energy of 38 nJ and a 1.25-NA oil-immersion objective was used to obtain a desired grating strength of 21.67 dB. The top-view morphology and corresponding transmission and reflection spectra are shown in Figure 4a,b, respectively. It is clear that the 0.535 μm periodic highly localized RIMs are visible, and thus a first-order FBG is effectively achieved. Notably, the size of the focal spot is determined by the diffraction limit (*D_a_ = 1.22λ*/NA, where *D_a_* is the diameter of the focal spot, *λ* is the laser wavelength, and NA is the numerical aperture of the objective. As for the 513 nm *fs* laser and 1.25-NA objective employed in our experiments, one can calculate that the spot size is ~501 nm. Additionally, due to the effect of nonlinear multiphoton absorption, the spatial resolution of *fs* laser nanofabrication could be further improved [24]. Thus, a grating period with a spatial resolution of 535 nm could be achieved. However, it is worth noting that excessively high intensity pulse energy will lead to overlapping RIMs in the first-order FBG because of the severe damage of RIMs, leading to undesired spectral distortions. Thus, an appropriate control of pulse energy is necessary for the fabrication of first-order FBGs. Compared with the second-order FBG in Figure 3b, the first-order FBG showed in Figure 4b exhibits a similar grating strength. Importantly, the first-order FBG shows a significantly lower IL of 0.08 dB. For the fixed grating length, a lower pulse energy was required to achieve a particular grating strength for the formation of a first-order FBG, since the number of grating pitches doubled, thereby balancing the refractive index modulation Δ*n*. In this case, the RIMs in the fiber core induced by the lower *fs* pulse energy are smoother, and thus reduce the insertion loss [25]. Therefore, in the case of carefully tailoring the pulse energy, first-order FBGs are more promising for achieving high quality FBGs.

### 3.3. Optimization of High-Quality FBG

**Figure 5:**
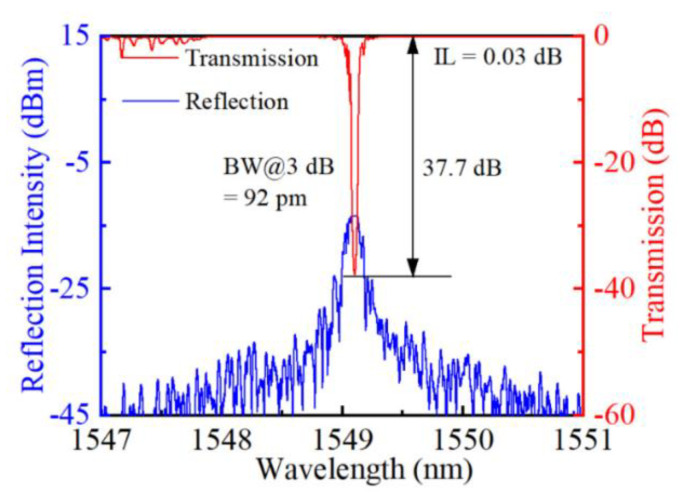
Transmission and reflection spectra of the inscribed 40 mm long first-order FBG.

According to the results and analysis above, the spectral characteristics of FBGs are dependent on the geometry of the RIM region and grating order. Moreover, the reflectivity *R* of an FBG is expressed in terms of the coupling strength coefficient *κ* and grating length *L*, as expressed in equation (2). The IL, resulting from the photo-induced damaged RIMs, is highly correlated to the intensity of the *fs* pulse energy. Thus, with appropriate control on pulse energy and grating length, one could expect further improvement in spectral quality. Herein, a 40 mm-long grating was inscribed using a translation speed of 0.535 mm/s, leading to a first-order FBG with a period of 0.535 μm and a Bragg wavelength of 1549.2 nm. The pulse energy for inscribing the FBG was set as 19 nJ to obtain smooth RIMs. The spectrum of the inscribed FBG was shown in Figure 5. We could observe a lower IL of only 0.03 dB, a 3 dB bandwidth of 92 pm and a dip resonance of 37.7 dB in transmission, corresponding to a high reflectivity of more than 99.9%. Typically, the ratio of the coupling strength coefficient to the scattering loss coefficient *κ/α* is a crucial parameter of high-quality FBGs. The coupling strength coefficient *κ* and scattering loss coefficient *α* can be expressed as [26]:

*κ* = *ln* (*T_B_*)/(*−2L*), *and α = ln* (*T_IL_*)/(*−2L*) (5)

where *T_B_* is the Bragg resonance attenuation, *T_IL_* is the insertion loss, and *L* is the grating length. Significantly, the *κ/α* of the inscribed 40 mm-long first-order FBG is 1459, which is much higher than the FBGs fabricated by the core scanning method [14]. It is worth noting that the spectral performance of the inscribed FBG is comparable to those fabricated by phase-mask technologies (i.e., transmission dip 21.08 dB insertion loss 0.08 dB) [27].

### 3.4. FBGs with Various Bragg Wavelengths

**Figure 6:**
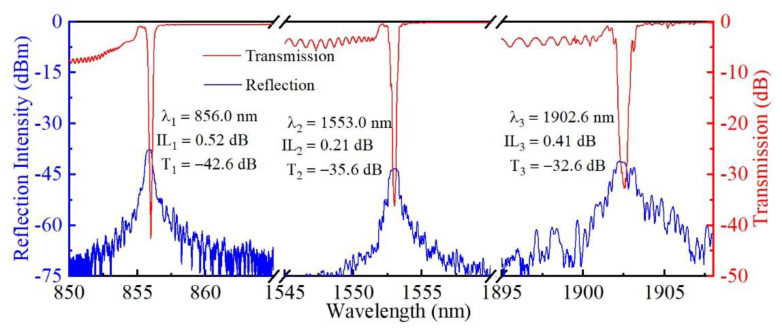
Transmission and reflection spectra of three inscribed FBGs with various Bragg wavelengths.

The *fs* laser PBP technology exhibits a significant advantage of excellent wavelength flexibility, since the FBG period can be easily tuned by controlling the *fs* pulse frequency and translation velocity of the fiber during the inscription process. Therefore, Bragg resonances could be controlled within the desired wavelength bands. For illustration, three 4 mm-long gratings were inscribed in a section of thin-core SMF (CS980/125-16/250) and two sections of standard SMF using a translation speed of 0.592, 1.072, and 1.316 mm/sec, respectively, leading to three second-order FBGs (i.e., S1, S2 and S3) with periods of 0.592, 1.072 and 1.316 μm. Identical pulse energy of 70 nJ was used for inscribing S1-S3. As shown in Figure 6, three FBGs with Bragg wavelengths of 856.0, 1553.0, and 1902.6 nm are achieved, with dip resonances of −42.6, −35.6, and −32.6 dB and insertion loss of 0.52, 0.21, and 0.41 dB, respectively. Note that the intensity of Bragg resonance reduces as the grating period increases, which results from the reduction of grating pitch quantities. Additionally, the FBG with a higher central wavelength showed a wider bandwidth [28]. Thus, the FBG with a desired Bragg wavelength and resonance strength could be flexibly tailored, thus allowing further applications of FBGs in arbitrary wavelength ranges.

**Figure 7:**
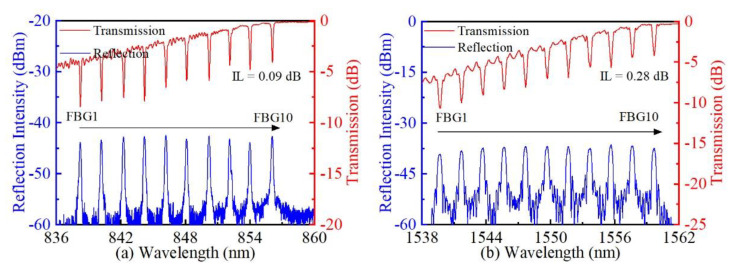
Transmission and reflection spectra of FBG WDM arrays consisting of 10 FBGs with Bragg wavelengths ranging (**a**) from 838 to 856 nm and (**b**) from 1538 to 1562 nm.

Additionally, two WDM FBG arrays with various Bragg wavelengths were successfully fabricated in a thin-core SMF (YOFC, CS980/125-16/250, Wuhan, China) and standard SMF, respectively. Ten 4 mm-long FBGs (i.e., FBG1, FBG2, …, FBG10) were gradually inscribed into a thin core SMF with translation speeds ranging from 0.575 to 0.584 mm/s with a step of 1 µm/s, achieving a WDM FBG array with 10 Bragg wavelengths ranging from 838 to 856 nm (i.e., WDM1). The spacing between two adjacent FBGs is 2 mm along the fiber axis. A 20 nJ pulse energy was used for inscribing the WDM1. In the same way, another WDM FBG array (i.e., WDM2) with Bragg wavelengths ranging from 1538 to 1562 nm was inscribed in a standard SMF using a 24 nJ pulse energy. Figure 7a,b shows the spectra of WDM1 and WDM2, respectively. Transmission and reflection spectra were measured, while the broadband light was input from the FBG1 end to the FBG10 end. It could be seen from the transmission of Figure 7a,b that the dip resonance of each FBG varies from −4 to −5 dB, corresponding to reflectivity from 61 to 68%. Importantly, the insertion losses of WDM1 and WDM2 are as low as 0.09 and 0.28 dB, respectively. Moreover, we could observe from the reflection that each FBG shows relatively uniform peak intensity. In particular, the high SNR peak and uniform reflectivity are more beneficial for simultaneous demodulation in sensing. Thus, such high-quality WDM FBG arrays could be further applied to quasi-distributed optical fiber sensors.

### 3.5. Birefringence-Tunable FBG-FP

Generally, FBGs inscribed by using *fs* laser PbP technology exhibit high birefringence, resulting from the laser-induced asymmetric and elliptical RIMs in the fiber core. This property could be exploited for polarization-selection in fiber lasers. For illustration, a FBG Fabry-Perot (FBG-FP) cavity was fabricated in a section of SMF by inscribing two 2 mm-long uniform first-order FBGs with a spacing of 1 mm along the fiber axis. The two inscribed FBGs have the same period of 0.535 μm, and the on-target single pulse energy used for fabricating each FBG was 37 nJ. The polarization-resolved transmission spectra were measured by a Muller-matrix-based commercial polarization analysis system that consists of a tunable laser (Keysight, N7776C, Santa Rosa, CA, USA), a polarization synthesizer (Keysight, N7788C, Santa Rosa, CA, USA), and an optical power meter (Keysight, N7744A, Santa Rosa, CA, USA) with a resolution of 0.3 pm.

**Figure 8:**
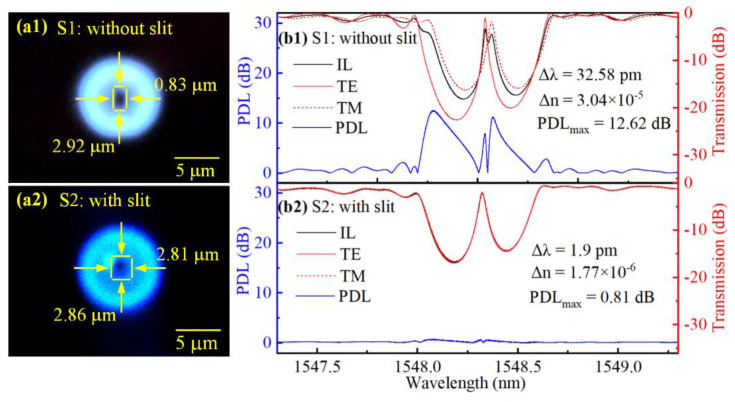
Two FBG-FPs inscribed without slit beam shaping (i.e., S1) and with slit beam shaping (i.e., S2). (**a**) Cross-sectional-view microscope images and (**b**) corresponding spectra of transmission, two orthogonal linear polarization modes (TE and TM), and PDL.

Figure 8a1 shows the cross-sectional-view microscope image of a RIM, which is an ellipse with a major axis and a minor axis length of 2.92 and 0.83 μm, respectively. As shown in Figure 8b1, we can observe that only one resonance peak arises within the stopband width, corresponding to a single longitudinal mode within the resonator cavity. Note that polarization splitting arises in the resonance peak. This indicates the existence of an evident refractive index difference between the orthogonal polarization modes resulting from the high birefringence. The birefringence of the first-order FBGs can be calculated from Δ*n_B_* = Δ*λ/2Λ*, where Δ*λ* is the Bragg wavelength difference between two orthogonal polarization modes (i.e., TE and TM), *Λ* is the period of grating. The FBG-FP cavity exhibits a Δ*λ* of 32.58 pm, corresponding to a high Δ*n_B_* of 3.04 × 10^−5^. Moreover, the difference in transmission resonance attenuation between TE and TM modes, denoted by Δ*T* in Figure 8, reaches ~6.3 dB, and the PDL is up to 12.62 dB. Thus, the TE polarization mode would experience a stronger intra-cavity reflectivity and lower loss, thus leading to a more dominant polarization mode operation than the TM polarization mode. This could be the key mechanism of polarization-discrimination for fiber lasers. Therefore, we could deduce that distributed Bragg reflector (DBR) fiber lasers operated in single longitudinal mode and single polarization could be achieved in gain fibers by such a high-birefringence FBG-FP cavity.

On the contrary, the polarization splitting would be a disadvantage when the FBG-FP cavity serves as a narrow-band filter or a sensor based on the narrow transmission band, since the high birefringence could lead to a higher bit error rate and reduced sensing accuracy. To address this, the slit beam shaping method was used for inscribing an FBG-FP based on the *fs* laser PbP technology. The slit width was set as 0.8 mm and the on-target single pulse energy was 86 nJ. The FBG-FP consisted of two 2 mm-long uniform first-order FBGs with a spacing of 1 mm. Figure 8a2 shows a cross-sectional view of the FBG-FP, a near-circular pattern with a major axis and a minor axis length of 2.86 and 2.81 μm, respectively. The polarization-resolved transmission spectra of the FBG-FP are shown in Figure 8b2. Note that the peak within the stopband shows a 3 dB bandwidth of 35 pm without polarization splitting. Moreover, the FBG-FP exhibits a Δ*λ* of 1.9 pm, corresponding to a low Δ*n_B_* of 1.77 × 10^−6^. The difference in transmission resonance attenuation Δ*T* is ~0.3 dB, and the PDL is as low as 0.81 dB. Compared with the FBG-FP without slit beam shaping, the FBG-FP with slit beam shaping shows a significantly lower birefringence due to the near-circular cross-sectional pattern induced by the shaping *fs* laser beam. Such a low-birefringence FBG-FP is valuable in the fields of fiber-optic telecommunications and high-accuracy fiber-optic sensing. In addition, the ellipticity of the cross-sectional pattern in RIMs could be further adjusted by changing the slit width. Thus, it is promising to achieve birefringence-tunable FBG-FPs with the slit beam shaping method for *fs* laser PbP technology.

## 4. Conclusions

We have further demonstrated the inscription of high-quality FBGs with high reflectivity and low insertion loss based on *fs* laser PbP technology. The effects of the focusing geometry, grating order, laser energy and grating length on the spectral response were experimentally investigated. With the optimized parameters, a 40 mm-long high-quality first-order FBG with a reflectivity >99.9% and insertion loss of 0.03 dB was successfully created. The corresponding ratio of coupling strength coefficient to the scattering loss coefficient is significantly improved to 1459. Subsequently, three FBGs with Bragg wavelengths of 856, 978.6, and 1902.6 nm were obtained, and two low-loss WDM FBG arrays consisting of 10 FBGs were realized, with Bragg wavelengths ranging from 838 3 to 856 nm and 1538 to 1562 nm. Moreover, birefringence-tunable FBG-FP cavities were realized by using a slit beam shaping method for *fs* laser PbP technology. The birefringence of the inscribed FBG-FP cavity with slit beam shaping was reduced to 1.77 × 10^−6^. Therefore, the high-quality FBGs created by *fs* laser PbP technology are promising in the applications of communications, sensing and lasers.

## Data Availability

All the data presented in this study are available in this article.
